# Positive Effect of Air Purifier Intervention on Baroreflex Sensitivity and Biomarkers of Oxidative Stress in Patients with Coronary Artery Disease: A Randomized Crossover Intervention Trial

**DOI:** 10.3390/ijerph19127078

**Published:** 2022-06-09

**Authors:** Sang-Yong Eom, Aryun Kim, Ju-Hee Lee, Sang Min Kim, Sang-Yeub Lee, Kyung-Kuk Hwang, Hyun-Joung Lim, Myeong-Chan Cho, Yong-Dae Kim, Jang-Whan Bae, Jun Hyung Kim, Dae-In Lee

**Affiliations:** 1 Department of Preventive Medicine, College of Medicine, Chungbuk National University, Cheongju 28644, Korea; esangy@cbnu.ac.kr (S.-Y.E.); ydkim@cbnu.ac.kr (Y.-D.K.); 2 Department of Neurology, Chungbuk National University Hospital, Cheongju 28644, Korea; mypioneer97@gmail.com; 3 Department of Cardiology, Chungbuk National University Hospital, Cheongju 28644, Korea; juheelee@chungbuk.ac.kr (J.-H.L.); masuri75@chungbuk.ac.kr (S.M.K.); louisahj@gmail.com (S.-Y.L.); kyungkuk@chungbuk.ac.kr (K.-K.H.); mccho@cbnu.ac.kr (M.-C.C.); drcorazon@hanmail.net (J.-W.B.); 4 Department of Internal Medicine, College of Medicine, Chungbuk National University, Cheongju 28644, Korea; 5 Division of Allergy and Respiratory Disease Research, Korea National Institute of Health, Cheongju 28159, Korea; hjlim1121@korea.kr; 6 Department of Internal Medicine, College of Medicine, Chungnam National University, Daejeon 34134, Korea

**Keywords:** particulate matter, air purifier, baroreflex sensitivity, oxidative stress, coronary artery disease

## Abstract

Exposure to fine particulate matter increases the risk of cardiovascular morbidity and mortality. Few studies have tested the beneficial effect of indoor air filtration intervention in patients with cardiovascular disease. The aim of this study is to investigate the effect of air filtration on mitigating cardiovascular health in patients with coronary artery disease. This randomized, double-blind, crossover study is conducted with 38 coronary artery disease patients. The intervention consists of the following three periods: two-week active and sham air filtration interventions, with a two-week washout period. The indoor PM_2.5_ concentration is continuously monitored during the entire study period. We measure the blood pressure, heart rate variability, baroreflex sensitivity, autonomic function test results, and endothelial function. The two-week active air filtration intervention for two weeks reduces the average indoor concentration of PM_2.5_ by 33.9%. The indoor PM_2.5_ concentration is significantly correlated to cross-correlation baroreflex sensitivity. Active air filtration is significantly associated with a decrease in the indicator of oxidative stress represented as 8-hydroxy-2′-deoxyguanosine. This study shows that a short-term air filtration intervention improved baroreflex sensitivity and might reduce oxidative stress in coronary artery disease patients. These findings suggest that the use of an air purifier could mitigate the recurrence of cardiovascular disease events in patients with coronary artery disease.

## 1. Introduction

A large number of previous studies revealed that short-term and long-term exposure to fine particulate matter (≤2.5 µm in diameter) (PM_2.5_) was related to an excessive increase in the incidence of acute coronary syndrome and cardiac mortality [1,2,3]. The pathophysiological mechanism of PM_2.5_, which mediates cardiovascular responses, includes complex events from pollutant inhalation to end-organ effects. The inhalation of PM_2.5_ triggers oxidative stress, inflammation, and ion channel activation in the lungs [4,5]. Thereafter, PM_2.5_ can lead to impaired vascular and endothelial function, plaque instability, thrombosis, and atherosclerosis through impaired autonomic function and biological intermediates, which can lead to the development of coronary artery disease (CAD) [4,5]. Considering a global exposure mortality model showing the linear relationship between PM_2.5_ and cardiac mortality [6], active measures to reduce an individual’s exposure to PM_2.5_ are expected to lead to clear cardiovascular benefits. Although the establishment of a policy to reduce the total amount of air pollutants and its active implementation is the ultimate solution to the impact that PM_2.5_ has on health [7,8], a mitigating intervention can provide an immediate protective effect on health. Moreover, as 80% of humans’ time in a day can now be spent indoors, interventions to reduce indoor PM_2.5_ exposure have become more important.

While previous studies have suggested that air purifiers improve endothelial function [9,10], biomarkers related to inflammation and thrombosis [9,10,11,12,13], and blood pressure [11,12,13], there is a problem with applying such results to vulnerable patients with coronary artery disease. Additionally, the intervention effect that air filtration has on autonomic nervous system function, which plays an important role in the pathophysiologic effects of PM_2.5_ on health, has not been properly verified.

In the present study, we aimed to evaluate the subclinical cardiovascular effects of air purifiers on pathophysiological mechanisms in patients with coronary artery disease.

## 2. Materials and Methods

### 2.1. Study Participants

A total of 40 patients aged 55–80 years with coronary artery disease were recruited in this study. All of the study participants underwent percutaneous coronary intervention (PCI) and visited the outpatient clinic at the Cardiovascular Department of Chungbuk National University Hospital or Chungnam National University Hospital in South Korea. During the recruitment process, the exclusion criteria were as follows: patients with a left ventricular ejection fraction of <45%, decreased creatinine clearance <15 mL/min, chronic obstructive pulmonary disease (Stage III–IV), patients who underwent PCI within three months before the study, patients who experienced malignant arrhythmia (e.g., ventricular tachycardia or fibrillation), and patients who experienced cerebral infarction or cerebral hemorrhage within three months before the study.

### 2.2. Study Design

This randomized, three-period crossover intervention study was conducted between November 2020 and February 2021. The three periods included two-week active and sham filtration periods separated by a two-week washout period. The order of the active filtration and sham filtration period was assigned according to the crossover designed allocation table. In the active filtration period, an air-purifier system (model name: ACK 13OZOSKBR; clean air delivery rate of 324 m^3^/h) was operated with a high-efficiency particulate arrestance filter (H13), and it was operated without the filter in the sham period. The air purifier system was installed in the center of the main living room before the intervention, and it operated continuously after the intervention started. To enable blinding from the assignments, the air purifier system was always on, regardless of whether the active or sham period was underway during the intervention period, and the patients were instructed not to disassemble or operate the air purifier. All of the participants were instructed to wear a Korean filter 94 (KF94) mask in all spaces except in their homes. They were provided with these KF94 masks during the study period. The KF94 mask has been approved by the Korea Food & Drug Administration as the Korean standard for filtering facepiece respirators, and it has a similar filtering efficiency to the N95 mask.

All of the participants visited the hospital a total of four times (one day before the start and end of each intervention period (active and sham filtration)). The participants were asked to complete questionnaires during the first visit to obtain information regarding patient demographics, including age, sex, smoking, and medical history. Blood pressure measurements, autonomic function tests, flow-mediated dilatation tests, and biological samples were assessed during each visit.

The study was registered at the Clinical Research Information Service (https://cris.nih.go.kr/cris, accessed on 23 January 2020 registration number KCT0006572), an online registration system for clinical studies in Korea and one of the primary registries of the World Health Organization International Clinical Trials Registry Platform.

### 2.3. Indoor and Outdoor Particulate Matter Assessments

During the complete study period (six weeks), indoor PM_10_ and PM_2.5_ concentrations were continuously measured every minute using an internet of things (IoT)-based indoor air quality monitoring system (ADT-1783, Smart-Aircok, Seoul, Korea), and the information regarding the indoor PM_10_ and PM_2.5_ concentrations was sent to the central server. This system was installed at a distance of 1 m away from the air purifier system. Outdoor ambient PM_10_ and PM_2.5_ concentrations within the same period were obtained from an air monitoring station adjacent to the participant’s residence from the Ministry of Environment in Korea. To validate the PM measurements in this study, an IoT-based air quality monitoring system was installed outdoors (on the veranda in the building) and then compared with ambient PM_2.5_ measurement values from the nearby air monitoring station using the beta-ray absorption method. PM_2.5_ measurements that were obtained from the air quality monitoring system in this study were highly correlated with the ambient PM_2.5_ concentration obtained from a nearby air monitoring station (R^2^ = 0.904) (Figure S1).

### 2.4. Health Measurements

All of the participants were instructed to avoid caffeine, smoking, and alcohol one day before visiting the clinic and to fast for at least four hours beforehand. During each clinical visit, the health indicators in the present study were calculated in the following order: (1) blood pressure measurement; (2) heart rate variability measurement; (3) autonomic function test; (4) flow-mediated dilation; (5) blood sampling.

After resting for 5 min in a sitting position with their feet flat on the floor, the participant placed their upper arm at the level of their heart. An appropriate cuff that could cover 22–24 cm in length and 40% of the circumference of the upper arm was applied. The participant’s systolic blood pressure (SBP) and diastolic blood pressure (DBP) were measured using a mercury sphygmomanometer. During this procedure, the blood pressure and heart rate were validated on a finometer.

The participants lay down on a bed; heart rate variability was recorded for 5 min using Finapres Nova (V1.9.A.R5503, Finapres Medical System, Amsterdam, The Netherlands). Immediately after measuring heart rate variability, cross-correlation baroreflex sensitivity (xBRS) was calculated as an index of arterial baroreflex sensitivity following the method used in a previous study by Chun et al. [14]. Then, the autonomic function tests, including the deep breathing test, the Valsalva maneuver, and the head-up tilt test, were performed in that order. The protocol of the procedures and analysis for the autonomic function tests followed the methodology developed by Low et al. and Novak [15,16]. The baroreflex sensitivity (BRS) indexes, sympathetic indexes, and pressure recovery time were determined from the Valsalva maneuver. The flow-mediated vasodilation (FMD) test, including endothelium-dependent and endothelium-independent vasodilation, was performed. The FMD test was performed according to the protocol proposed by Deanfield [17]. Finally, blood sampling for C-reactive protein (CRP), Interleukin-6 (IL-6), and brain natriuretic peptide (BNP) was carried out in a commercial laboratory (Green Cross Medical Laboratory, Korea). Urinary 8-hydroxy-2′-deoxyguanosine (8–OHdG) was analyzed using a commercial enzyme-linked immunosorbent assay kit, following the manufacturer’s instructions (KOG-200S/E, Japan Institute for the Control of Aging, Shizuoka, Japan). The urinary 8-OHdG concentration was adjusted to the urinary concentration of creatinine to control for the variability in urine dilution. The detailed method used for the autonomic function and FMD tests is described in detail (File S1).

### 2.5. Statistical Analysis

The number of patients required to carry out the clinical trial was calculated based on changes in the mean DBP due to the use of air purifiers in a previous study [12], and it was calculated that 40 participants were needed to provide 80% power to detect a 4.8% difference in DBP with the crossover design.

The normality of the data was checked using the Shapiro-Wilk test. All of the data concerning health outcomes and biomarkers were transformed to a log-normal distribution, as the distribution was skewed. Statistical comparisons of the means of various variables were performed using the paired *t*-test. Linear mixed-effect models were used to determine the associations between repeated measurements for health outcomes or biomarkers and intervention modes. For mixed-effects models, we used an unstructured covariance matrix and entered the intervention model as a fixed effect and patients as a random effect to account for repeated measurements. Each model included age, sex, smoking status, house area, hypertension status, diabetes status, medication use (beta-blocker and angiotensin-converting enzyme inhibitor), sequence, and period as fixed covariate effects. All *p*-values were two-sided, and the statistical significance was set at <0.05. Statistical analyses were performed using Statistical Package for the Social Sciences (SPSS) software version 24.0 (IBM, Armonk, NY, USA).

## 3. Results

**Figure 1:**
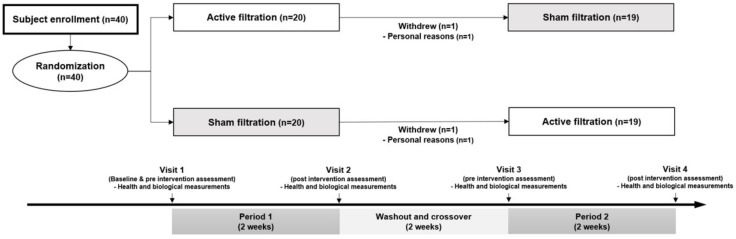
Flow chart of crossover trial. This randomized controlled trial consisted of three periods, two-weeks active and sham filtration interventions with two-week washout period.

An overall flow diagram showing the intervention process and exclusions was created (Figure 1). Of the 40 patients enrolled, 38 completed the 6 weeks of intervention sessions and were included in the final analyses. The mean age was 65.8 ± 6.4 years old, and 78.9% of the patients were female (Table 1). Some patients were diagnosed with ST-segment elevation myocardial infarction (44.7%), angina (39.5%), and non-ST segment elevation myocardial infarction (15.8%). The prevalence of hypertension or diabetes mellitus was 34.2%, and 68.4% of the patients were treated with beta-blockers. Of the total patients, the proportion of current smokers was 21.1%, and 34.2% of them were passive smokers. The baseline characteristics of the study subjects by intervention sequence are presented in Table S1.

The indoor PM_10_ concentration in the active filtration phase (18.6 ± 10.6 µg/m^3^) was lower than in the sham filtration phase (27.4 ± 18.2 µg/m^3^). The indoor PM_2.5_ decreased to 12.3 µg/m^3^ in the active filtration phase as opposed to in the sham filtration phase. The indoor concentrations of PM_10_ and PM_2.5_ in the active filtration phase were 32.1% and 33.9% lower than those in the sham filtration phase, respectively (Table 2).

Before adjustment for clinical variables, no significant changes were observed in the DBP and heart rate in both intervention phases (Table 3). However, the SBP marginally decreased in the post-air purifier phase but not in the control phase. After the air purifier intervention, xBRS was significantly increased compared to the levels recorded in the pre-intervention phase, but this increase was not observed in the control phase. Urinary 8-OHdG concentration, which is an indicator of oxidative stress, increased significantly in the control phase, but an insignificant decrease was observed in the air purifier phase. Markers for the deep breathing test, tilt table test, flow-mediated vasodilation, and inflammation did not present differences between the pre- and post-intervention phases (Table S2). Differences in health outcomes between the pre- and post-intervention phases were not statistically significant (Table S3).

The percent changes in selected health outcomes, according to the intervention or the indoor PM_2.5_ level, are shown using a linear mixed model (Table 4). After adjusting for age, sex, smoking status, house area, hypertension status, diabetes status, medication use, sequence, and period, the urinary 8-OHdG level was significantly reduced in the active filtration phase compared to the sham filtration phase (−15.59%, 95% CI: −25.38 to −4.45). A one percent increase in the indoor PM_2.5_ concentration significantly decreased the xBRS by −19.94% (95% CI: −39.29 to −0.59). However, the blood pressure and inflammatory markers were not changed by the intervention phase or the indoor PM_2.5_ level. Other cardiovascular outcomes (i.e., the deep breathing test, tilt table test, and flow-mediated vasodilation) also did not show significant associations with the intervention or indoor PM_2.5_ level (Table S4).

## 4. Discussion

The main findings of the present study are as follows: First, the intervention with the air purifier reduced the average indoor PM_2.5_ concentration by 33.9% compared to that of the control mode. Second, the application of an air purifier for two weeks reduced the urinary 8-OHdG level by 15%, although there was no statistical significance between the indoor PM_2.5_ concentration and the urinary 8-OHdG level. Third, after adjusting the clinical variables, the xBRS was negatively correlated to the indoor concentration of PM, which tended to increase due to the air purifier. Fourth, the present study suggests that fine PM has a null effect on endothelial function and blood pressure in CAD patients.

There is abundant evidence that air pollution contributes to the increase in the incidence of cardiovascular morbidities and mortality [1,2,3,4,5,6,8,18]. A meta-analysis of seven studies revealed that 0.35% (95% CI 0.06–0.65%) excess mortality of cardiovascular death was proportional to each 10 µg/m^3^ increase in short-term exposure to PM_2.5_ [18]. Chronic exposure studies showed that each 10 µg/m^3^ increment in PM_2.5_ was associated with an additional increase in cardiovascular mortality, ranging from 15 to 31% [19,20]. Additionally, a recent study revealed that non-fatal myocardial infarction (MI) increased by 2.5% with each 10 µg/m^3^ increase in PM_2.5_ [21].

The association of 8-OHdG as an indicator of reactive oxygen stress with CVD events has already been reported in previous studies [22]. Nagayoshi et al. revealed that MI patients had a higher level of 8-OHdG before undergoing reperfusion therapy compared to normal individuals [22].

Moreover, a high correlation between the total urinary 8-OHdG level and infarcted size in patients with MI (r = 0.87 and *p* < 0.01) and recurrent ischemic stroke and cardiac death were suggested [23,24]. Therefore, a 15% reduction of 8-OHdG levels in patients who underwent the active filtration period compared to the sham filtration period implies that this intervention using an air purifier can effectively alleviate the recurrence and exacerbation of coronary artery disease. Moreover, this finding is consistent with a previous study by Chuang et al. [25], which proved the positive effect that air filtration had on the decrease in reactive oxygen species activity, measured using 8-OHdG. However, this finding deserves more attention because the present study is the first study that was conducted with vulnerable patients, such as CAD patients.

However, the indoor PM_2.5_ concentration was not significantly associated with the urinary 8-OHdG level in this study. This result may be due to the fact that not only PM_2.5_ but also other indoor pollutants with oxidative potential (i.e., gaseous phase pollutants, such as volatile organic compounds) were removed by the air purifier. In this context, it is rational that the intervention effect of the air purifiers was evaluated through integrated air quality improvement rather than a reduction in the PM_2.5_ concentration. Therefore, our results provide the limited presumption that improvement in indoor air quality using an air purifier is effective in lowering oxidative stress in CAD patients.

For the first time in a human study, we demonstrated an improvement in BRS associated with short-term reductions in indoor PM_2.5_ concentrations. Although the mechanism of the activation of pulmonary receptors via PM_2.5_ results in autonomic nervous system imbalance, leading to CVD events, which has been explained as one of the major pathways [2,4,18], the association between PM_2.5_ and baroreflex sensitivity was very limited in two animal experiments with conflicting results [26,27]. As decreased BRS in MI patients significantly increased the malignancy of the event, including lethal arrhythmias and mortality in a large prospective registry [28], our findings in this study highlight the clinical importance of managing PM_2.5_ in CAD patients. However, in our study, it was confirmed that at a personal level, active filtration using an air purifier improved the BRS levels in the univariate analysis, but only statistical trends were confirmed in the multivariate analysis. This finding may be due to the fact that the size of the interventional study population was inadequate to evaluate the efficacy of the air purifier.

Most of the previous studies concerning the effect of air purifiers on health problems have focused on blood pressure response [12,13,29]. A recent meta-analysis based on 10 studies showed a significant reduction in mean SBP levels by 3.94 mmHg (95% CI, −7.00 to −0.89; *p* = 0.01) through a short-term intervention using an air purifier [29]. However, in this study, the effects of active air-filtration on blood pressure were not consistent with those of previous meta-analyses, which may be due to the difference in the study population between this study, in which the population had comorbidities, and previous studies, which targeted healthy people. Therefore, additional research is needed to evaluate whether the application of an air purifier in hypertensive patients has an additive effect on reducing their blood pressure with antihypertensive drugs.

The different effects of an air purifier on endothelial function in our study (which presented a null result) and previous studies (which presented positive effects) can be explained in the same way. Two previous studies by Brauner et al. and Allen et al. demonstrated that active air filtration increased the reactive hyperemia index, causing an increase in endothelial function by 8–9.4% in healthy individuals compared to a sham air-filtration period [9,10]. However, our study was conducted with patients who took medications, such as nitrates and statins, which influence endothelial function; thus, the endothelial function may not have been additively improved by air purification.

This study is the first to evaluate the effect of PM_2.5_ on vulnerable patients with CAD. Additionally, it is worth noting that the present study analyzed the effect of air filtration on cardiovascular response in a real situation without stopping the use of cardioactive drugs or smoking in CAD patients. However, despite these strengths, there were some limitations. First, this study involved a relatively small sample size that may have reduced the statistical power for detecting improvements in cardiovascular response. Secondly, although we randomly assigned the intervention sequence, the concentration of ambient PM_2.5_ during the air purifier intervention period was higher than that during the control intervention period. Therefore, the patients’ outdoor activity time may have influenced the intervention effect. However, as this study was conducted during the COVID-19 outbreak, all of the patients involved wore a Korean filtration mask with 94% filtration efficacy, which was provided by our research team, in all spaces outside their homes. The effect of air filtration was tested with the minimized effect of outdoor PM_2.5_.

## 5. Conclusions

In this study, the use of an air purifier for two weeks improved depressed BRS and might attenuate oxidative stress levels in patients with CAD. These findings suggest that a measure used indoors may effectively improve the health of patients with CAD. Moreover, this study suggests the necessity for further studies over longer time frames and in environments with higher PM_2.5_ concentrations.

## Figures and Tables

**Table 1:** Characteristics of study participants.

Variable		Mean ± STD or N (%)
Number		38
Age	yrs	65.8 ± 6.4
Sex	Male:female	8:30
Height	cm	165.1 ± 7.9
Weight	kg	69.1 ± 8.7
BMI	kg/m^2^	25.4 ± 3.1
Clinical diagnosis	STEMI	17 (44.7)
	NSTEMI	6 (15.8)
	Unstable angina	15 (39.5)
Number of of prior PCI	1	34 (89.5)
	2	4 (10.5)
Underlying disease	Hypertension	13 (34.2)
	Diabetes	13 (34.2)
	Dyslipidemia	10 (26.3)
	HF	2 (5.3)
	TIA	1 (2.6)
Antianginal medication	Beta blocker	26 (68.4)
	ACEI/ARB	20 (52.6)
	Nitrate	7 (18.4)
Smoking status	Current smokers	8 (21.1)
	Secondary smokers	13 (34.2)

STEMI: ST-segment elevation myocardial infarction, NSTEMI: non-ST segment elevation myocardial infarction, HF: heart failure, TIA: transient ischemic attack, ACEI: angiotensin converting enzyme inhibitor, ARB: angiotensin receptor blocker, PCI: percutaneous coronary intervention, yrs: years.

**Table 2:** Particulate matter concentration in indoor and outdoor environments during the intervention periods.

		Intervention		*p*-Value
		Sham Filtration	Active Filtration	
PM_10_, µg/m^3^	Indoors	27.4 ± 18.2	18.6 ± 10.6	<0.001
	Outdoors	40.2 ± 7.9	43.3 ± 9.9	0.032
PM_2.5_, µg/m^3^	Indoors	18.6 ± 14.9	12.3 ± 8.0	<0.001
	Outdoors	23.4 ± 5.5	26.0 ± 7.2	0.007

PM: particulate matter, *p*-value was calculated using paired *t*-test.

**Table 3:** Changes of health outcomes between pre- and post-intervention according to intervention phase.

	Sham Filtration			Active Filtration		
	Pre	Post	*p*-Value	Pre	Post	*p*-Value
Blood pressure						
SBP (mmHg)	125.50 (2.10)	121.70 (2.19)	0.091	126.40 (1.90)	121.90 (2.31)	0.065
DBP (mmHg)	73.96 (1.26)	73.98 (1.52)	0.984	74.27 (1.36)	73.90 (1.61)	0.815
HR (beats/min)	63.30 (1.95)	63.86 (2.10)	0.590	63.36 (1.89)	64.79 (1.93)	0.266
Autonomic nerve function						
xBRS (ms/mmHg)	7.33 (0.88)	8.02 (0.81)	0.248	7.96 (0.70)	9.52 (0.74)	0.028
Inflammation and oxidative stress markers						
CRP (mg/dL)	0.52 (0.07)	0.51 (0.10)	0.469	0.59 (0.10)	0.68 (0.18)	0.132
IL-6 (pg/mL)	2.65 (0.27)	2.57 (0.28)	0.738	2.51 (0.27)	3.01 (0.44)	0.118
BNP (pg/mL)	30.07 (4.37)	31.55 (5.45)	0.616	24.25 (3.95)	25.55 (4.80)	0.518
8-OHdG (µg/g creatinine)	7.63 (0.76)	8.58 (0.68)	0.016	7.59 (0.64)	7.15 (0.83)	0.431

Data presented as geometric mean (geometric standard error); *p*-value was calculated using paired *t*-test. SBP: systolic blood pressure, DBP: diastolic blood pressure, HR: heart rate, xBRS: cross-correlation baroreflex sensitivity, CRP: C-reactive protein, IL: interleukin, BNP: brain natriuretic peptide, 8–OHdG: 8-hydroxy-2′-deoxyguanosine.

**Table 4:** Percent changes in blood pressure, inflammation, and oxidative stress markers levels according to intervention phases or change in indoor PM_2.5_.

Outcomes	Intervention (Active vs. Sham)			Indoor PM_2.5_ Level (One Percent Increase)		
	Percent, %	95% CI	*p*-Value	Percent, %	95% CI	*p*-Value
Blood pressure						
SBP (mmHg)	−0.71	−4.17, 2.87	0.736	−1.25	−6.38, 3.87	0.622
DBP (mmHg)	−0.32	−3.86, 3.34	0.880	−2.51	−7.97, 2.95	0.356
HR (beats/min)	0.50	−3.91, 5.10	0.853	−3.38	−8.59, 1.83	0.196
Autonomic nerve function						
xBRS (ms/mmHg)	14.44	−2.67, 34.57	0.168	−19.94	−39.29, −0.59	0.044
Inflammation and oxidative stress markers						
CRP (mg/dL)	17.36	−15.68, 63.36	0.418	−8.60	−54.01, 36.82	0.703
IL-6 (pg/mL)	−5.61	−21.00, 12.77	0.587	20.10	−1.40, 41.59	0.066
BNP (pg/mL)	18.82	−1.91, 43.91	0.138	−1.15	−29.71, 27.40	0.935
8-OHdG (µg/g creatinine)	−15.56	−25.38, −4.45	0.027	3.35	−13.69, 20.40	0.692

Adjusted for age, sex, smoking status, house area, hypertension status, diabetes status, and medication use (beta-blocker, ACE inhibitor), sequence, and period. CI: confidence interval, SBP: systolic blood pressure, DBP: diastolic blood pressure, HR: heart rate, xBRS: cross-correlation baroreflex sensitivity, CRP: C-reactive protein, IL: interleukin, BNP: brain natriuretic peptide, 8-OHdG: 8-hydroxy-2′-deoxyguanosine.

## Data Availability

Not applicable.

## Supplementary Materials

The following supporting information can be downloaded at: https://www.mdpi.com/article/10.3390/ijerph19127078/s1, Table S1: Baseline characteristics of study participants by intervention sequence, Table S2: Changes in heart rate variability, deep breathing test, tilt table test, and flow-mediated vasodilation between pre- and post-intervention according to intervention phase. Table S3: Differences in health outcomes between pre- and post-intervention according to intervention phase. Table S4: Percent changes in deep breathing test, tilt table test, and flow-mediated vasodilation according to intervention phases or change in indoor PM_2.5_. Figure S1: Comparison of outdoor PM_2.5_ concentration between air pollution monitoring station and air quality measurement device used in this study. File S1: Study protocol.

## References

[1] Burnett R.T., Pope C.A., Ezzati M., Olives C., Lim S.S., Mehta S., Shin H.H., Singh G., Hubbell B., Brauer M. (2014). An integrated risk function for estimating the global burden of disease attributable to ambient fine particulate matter exposure. Environ. Health Perspect..

[2] Lu F., Xu D., Cheng Y., Dong S., Guo C., Jiang X., Zheng X. (2015). Systematic review and meta-analysis of the adverse health effects of ambient PM2.5 and PM10 pollution in the Chinese population. Environ. Res..

[3] Pope C.A., Dockery D.W. (2006). Health effects of fine particulate air pollution: Lines that connect. J. Air Waste Manag. Assoc..

[4] Al-Kindi S.G., Brook R.D., Biswal S., Rajagopalan S. (2020). Environmental determinants of cardiovascular disease: Lessons learned from air pollution. Nat. Rev. Cardiol..

[5] Hamanaka R.B., Mutlu G.M. (2018). Particulate matter air pollution: Effects on the cardiovascular system. Front. Endocrinol..

[6] Burnett R., Chen H., Szyszkowicz M., Fann N., Hubbell B., Pope C.A., Apte J.S., Brauer M., Cohen A., Weichenthal S. (2018). Global estimates of mortality associated with long-term exposure to outdoor fine particulate matter. Proc. Natl. Acad. Sci. USA.

[7] Dominici F., Peng R.D., Zeger S.L., White R.H., Samet J.M. (2007). Particulate air pollution and mortality in the United States: Did the risks change from 1987 to 2000?. Am. J. Epidemiol..

[8] Yorifuji T., Kashima S., Doi H. (2016). Fine-particulate Air Pollution from Diesel Emission Control and Mortality Rates in Tokyo: A Quasi-experimental Study. Epidemiology.

[9] Brauner E.V., Forchhammer L., Moller P., Barregard L., Gunnarsen L., Afshari A., Wåhlin P., Glasius M., Dragsted L.O., Basu S. (2008). Indoor particles affect vascular function in the aged: An air filtration-based intervention study. Am. J. Respir. Crit. Care Med..

[10] Allen R.W., Carlsten C., Karlen B., Leckie S., van Eeden S., Vedal S., Wong I., Brauer M. (2011). An air filter intervention study of endothelial function among healthy adults in a woodsmoke-impacted community. Am. J. Respir. Crit. Care Med..

[11] Shao D., Du Y., Liu S., Brunekreef B., Meliefste K., Zhao Q., Chen J., Song X., Wang M., Wang J. (2017). Cardiorespiratory responses of air filtration: A randomized crossover intervention trial in seniors living in Beijing: Beijing Indoor Air Purifier Study, BIAPSY. Sci. Total Environ..

[12] Chen R., Zhao A., Chen H., Zhao Z., Cai J., Wang C., Yang C., Li H., Xu X., Ha S. (2015). Cardiopulmonary benefits of reducing indoor particles of outdoor origin: A randomized, double-blind crossover trial of air purifiers. J. Am. Coll. Cardiol..

[13] Morishita M., Adar S.D., D’Souza J., Ziemba R.A., Bard R.L., Spino C., Brook R.D. (2018). Effect of Portable Air Filtration Systems on Personal Exposure to Fine Particulate Matter and Blood Pressure Among Residents in a Low-Income Senior Facility: A Randomized Clinical Trial. JAMA Intern. Med..

[14] Chun K.J., Yim H.R., Park J., Park S.J., Park K.M., On Y.K., Kim J.S. (2016). Role of Baroreflex Sensitivity in Predicting Tilt Training Response in Patients with Neurally Mediated Syncope. Yonsei Med. J..

[15] Low P.A. (2003). Testing the autonomic nervous system. Semin. Neurol..

[16] Novak P. (2011). Quantitative autonomic testing. J. Vis. Exp..

[17] Deanfield J.E., Halcox J.P., Rabelink T.J. (2007). Endothelial function and dysfunction: Testing and clinical relevance. Circulation.

[18] Fu P., Guo X., Cheung F.M.H., Yung K.K.L. (2019). The association between PM2.5 exposure and neurological disorders: A systematic review and meta-analysis. Sci. Total Environ..

[19] Krewski D., Jerrett M., Burnett R.T., Ma R., Hughes E., Shi Y., Turner M.C., Pope C.A., Thurston G., Calle E.E. (2009). Extended Follow-Up and Spatial Analysis of the American Cancer Society Study Linking Particulate Air Pollution and Mortality.

[20] Crouse D.L., Peters P.A., van Donkelaar A., Goldberg M.S., Villeneuve P.J., Brion O., Khan S., Atari D.O., Jerrett M., Pope C.A. (2012). Risk of nonaccidental and cardiovascular mortality in relation to long-term exposure to low concentrations of fine particulate matter: A Canadian national-level cohort study. Environ. Health Perspect..

[21] Mustafic H., Jabre P., Caussin C., Murad M.H., Escolano S., Tafflet M., Périer M.-C., Marijon E., Vernerey D., Empana J.-P. (2012). Main air pollutants and myocardial infarction: A systematic review and meta-analysis. JAMA.

[22] Nagayoshi Y., Kawano H., Hokamaki J., Miyamoto S., Kojima S., Shimomura H., Tsujita K., Sakamoto T., Yoshimura M., Ogawa H. (2005). Urinary 8-hydroxy-2′-deoxyguanosine levels increase after reperfusion in acute myocardial infarction and may predict subsequent cardiac events. Am. J. Cardiol..

[23] Mizukoshi G., Katsura K., Katayama Y. (2005). Urinary 8-hydroxy-2′-deoxyguanosine and serum S100beta in acute cardioembolic stroke patients. Neurol. Res..

[24] Brea D., Roquer J., Serena J., Segura T., Castillo J., Artico S. (2012). Oxidative stress markers are associated to vascular recurrence in non-cardioembolic stroke patients non-treated with statins. BMC Neurol..

[25] Chuang H.C., Ho K.F., Lin L.Y., Chang T.Y., Hong G.B., Ma C.M., Liu I.-J., Chuang K.-J. (2017). Long-term indoor air conditioner filtration and cardiovascular health: A randomized crossover intervention study. Environ. Int..

[26] Martin B.L., Thompson L.C., Kim Y.H., King C., Snow S., Schladweiler M., Haykal-Coates N., George I., Gilmour M.I., Kodavanti U.P. (2020). Peat smoke inhalation alters blood pressure, baroreflex sensitivity, and cardiac arrhythmia risk in rats. J. Toxicol. Environ. Health Part A.

[27] Carll A.P., Crespo S.M., Filho M.S., Zati D.H., Coull B.A., Diaz E.A., Raimundo R.D., Jaeger T.N.G., Ricci-Vitor A.L., Papapostolou V. (2017). Inhaled ambient-level traffic-derived particulates decrease cardiac vagal influence and baroreflexes and increase arrhythmia in a rat model of metabolic syndrome. Part. Fibre Toxicol..

[28] La Rovere M.T., Bigger J.T., Marcus F.I., Mortara A., Schwartz P.J. (1998). Baroreflex sensitivity and heart-rate variability in prediction of total cardiac mortality after myocardial infarction. Lancet.

[29] McDonald E., Cook D., Newman T., Griffith L., Cox G., Guyatt G. (2002). Effect of air filtration systems on asthma: A systematic review of randomized trials. Chest.

