# Supplementary material for: Positive Effect of Air Purifier Intervention on Baroreflex Sensitivity and Biomarkers of Oxidative Stress in Patients with Coronary Artery Disease: A Randomized Crossover Intervention Trial
Source: Int J Environ Res Public Health. 2022 Jun 9;19(12):7078. doi: 10.3390/ijerph19127078 (PMC9223013; doi:10.3390/ijerph19127078)
Supplement: Supplementary file 1 [file ijerph-19-07078-s001.zip › S1 Figure.pdf]

Figure S1

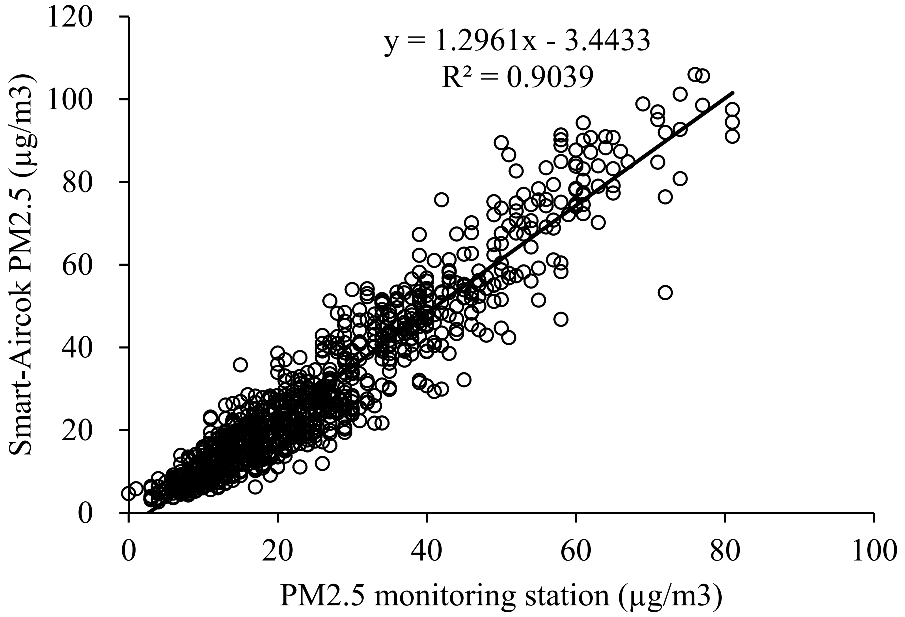

Figure S1. Comparison of outdoor PM2.5 concentration between air pollution monitoring station and air quality measurement device used in this study
