# Supplementary material for: Positive Effect of Air Purifier Intervention on Baroreflex Sensitivity and Biomarkers of Oxidative Stress in Patients with Coronary Artery Disease: A Randomized Crossover Intervention Trial
Source: Int J Environ Res Public Health. 2022 Jun 9;19(12):7078. doi: 10.3390/ijerph19127078 (PMC9223013; doi:10.3390/ijerph19127078)
Supplement: Supplementary file 1 [file ijerph-19-07078-s001.zip › S1 FILE (Study protocol).pdf]

Chungbuk National University Hospital	Clinical Investigation Plan Cover Page

Effect of the air-purifier on the hemodynamic alteration in patients with decreased cardio-pulmonary function
IRB Number: CBNUH 2019-11-006
CRIS Number: PRE20210105-007
Ver 3.0
Date: 21- AUG-2020

1. TABLE OF CONTENTS

1. TABLE OF CONTENTS**--2**

2. CLINICAL INVESTIGATION SYNOPSIS**--5**

3. INTRODUCTION**---9**

A. Background--9

B. Rationale for Intervention--11

C. Study Purpose and Objectives--11

i. Study Purpose---11

ii. Study Objectives---11

4. Methods**---11**

A. Study Participants, Design and Recruitment------------------------------11

i. Investigation Sites--11

ii. Study Population--12

iii. Study Design---12

iv. Sample Size Measures--13

v. Recruitment Procedures--13

vi. Patient Withdrawal--13

B. Criteria for Eligibility---14

i. Inclusion criteria--14

ii. Exclusion criteria--15

C. Intervention--15

D. Indoor air quality measurement--15

E. Study endpoints--15

i. Primary Endpoints---16

ii. Secondary Endpoints------16

F. Schedule of Activities------16

G. Assignment Consideration------17

i. Allocation------17

ii. Blinding------17

H. Study Procedures------17

i. Screening and Informed Consent Procedures------17

ii. Pre-treatment Procedures------18

iii. Intervention Procedure------18

iv. Wash-out Procedure------19

v. Measurement Protocol------20

I. Data Collection, management and Analysis------25

i. Data Collection------25

ii. Data Management------25

iii. Data Monitoring------26

iv. Statistical Methods------26

5. Patient Benefits and Risks**------26**

i. Potential Benefits to Study Subjects------26

ii. Potential Benefits to Study Subjects------27

iii. Clinical Risk Analysis------27

6. Adverse Events**------27**

i. Definitions------27

1. Serious Adverse Events------28

2. Serious Adverse Events------28

7. Ethics---**29**

i. Declaration of Helsinki--29

ii. Institutional Review Board Approval--29

iii. Informed Consent---29

iv. Patients Identification and Confidentiality---29

8. Regulatory Requirements--29

i. Compliance With Regulations Applicable to Clinical Trials------------------------30

ii. General Considerations---31

1. Discontinuation of the Study--31

2. Use of Information and Publication---31

2. CLINICAL INVESTIGATION SYNOPSIS

STUDY TITLE	Effect of improvement of in-door air quality (particulate matter) on the hemodynamic alteration In patients with decreased cardio-pulmonary function Using air-purifier
NAME AND LOCATION OF INVESTIGATION INSTITUTE	**Chungbuk National University Hospital**, 776, 1sunhwan-ro, Seowon-gu, Cheongju-si, Chungcheongbuk-do, Republic of Korea<br>**Chungnam National University Hospital,** 99, Daehak-ro, Yuseong-gu, Daejeon, Republic of Korea
NAME AND AFFLIATION OF RESEARCH DIRECTOR	**Dae-In Lee**, Clinical Associated Professor, Cardiologic Department of Chungbuk National University Hospital
Objectives	**(Final goal)**<br>Evaluation of the effect of air-purifier on hemodynamic changes according to air-purifier in the patients with coronary artery disease<br>**(Detailed goals)**<br>For patients with coronary artery disease<br>- Development of clinical research protocol for air-purifier intervention effect of air-purifier evaluation on hemodynamic function<br>- Evaluation of hemodynamic changes according to individual exposure levels to particulate matter<br>- Evaluation of the relevance of indoor particulate matter quality improvement and hemodynamic changes through indoor air quality intervention<br>- Threshold evaluation of the level of exposure to particulate matter that induces hemodynamic changes-<br>Prepared recommendations for the use of air-purifiers tailored to

	people with coronary artery disease
STUDY POPULATION	Among those over 40 years of age who are able to live a normal life with coronary artery disease (40 patients)
RESEARCH PERIOD	2 years from the date of IRB approval
CLINICAL RESEARCH DRUG	NONE
INCLUSION & EXCLUSION CRITERIA	● Inclusion criteria<br>55 years or older<br>– Under 85 years old<br>– Those who have had coronary angioplasty (40 patients)<br>– Left ventricular ejection fraction >45%<br>– Those who voluntarily signed and submitted the consent form<br>● Exclusion criteria<br>- Those who complain of symptoms of Functional class IV (NYHA class IV)<br>- Left ventricular ejection fraction < 15%<br>- Chronic obstructive pulmonary disease stage 3 ~ 4<br>- PCI within 3 months of enrollment<br>- Malignant arrhythmia (ventricular tachycardia or ventricular fibrillation) within 3 months of enrollment<br>- Cerebral infarction or hemorrhage within 3 months of enrollment
STUDY DESIGN	● Multicenter study: Chungbuk National University Hospital,

	Chungnam National University Hospital<br><br>● Randomized, crossover, interventional study<br><br>● Intervention period: 6 weeks<br><br>● Measurement of the total amount of particulate matter (PM 2.5) concentration<br><br>- Continuous measurement of indoor PM 2.5 concentration using IoT (Internet of Things)-based laser particulate matter measurement technology every 1 minute<br>- Calibration using a personal portable measuring device (light scattering method) and actual measurement of PM 2.5 concentration (gravimetric method) to secure the inspection reliability of the IoT measurement method<br>- Correction of confusion variables through living environment survey and personal time activity log<br><br>● Intervention of indoor air quality using an air-purifier<br><br>- HEPA Filter (H13 grade, 99.97% filtration rate for particulate matter over 0.3 um)<br><br>● Study procedures (crossover design including washout period)<br>- Intervention period (Phase 1): Operation of an air-purifier (active vs. sham filtration group with/without HEPA filter) in the living room for 2 weeks

	- Washout period (Phase 2): Washout for 2 weeks<br>- Intervention period (Phase 3): Repeat the experiment after crossing both groups<br>● Allocation of the order for intervention<br>- The order for active or sham filtration intervention was assigned accoring to the crossover designed allocation table
ASSESSMENT METHOD	● Main evaluation variable<br>- Total amount of particulate matter (PM2.5) by individual<br>- Vital sign (systolic blood pressure, diastolic blood pressure, pulse rate)<br>- Heart rate variability using Finapres NOVA<br>- Cross-correlation baroreflex sensitivity (Xbrs) using Finapres NOVA<br>- Autonomic nervous system function using Finapres NOVA<br>- Vascular endothelial cell function (brachial artery blood flow-mediated vasodilation through ultrasound)<br>- Blood biomarkers (biomarkers; hs-CRP, IL-6, BNP, etc.)
STATITISTICAL ANALYSIS	Analysis: Evaluation of hemodynamic changes according to the use of air-purifiers

3. INTRODUCTION

A. Background

In 2016, WHO announced that 4.2 million deaths were due to particulate matter (PM), especially vulnerable patients with heart disease, stroke, and chronic obstructive lung disease suffered health damage mainly from PM.

In 2004, the US Environmental Protection Agency (EPA) published the 'Integrated Science Assessment for Particulate Matter' on the causal relationship between particulate matter and disease through the consensus process of several subcommittees (animal test results, epidemiological investigations, clinical studies, socioeconomic factors).

An important implication of this publication is that, contrary to general expectations, the level of evidence of PM 2.5 on the health effects was highly proven in cardiovascular diseases (CVD), not respiratory or nervous system diseases. In addition, it was confirmed that PM 2.5 had a great effect on CVD patients in a short duration of several hours to several days.

As an important mechanism for the development of CVD, a reactive oxygen species-dependent pathway that propagates to terminal organ though pulmonary oxidative stress, systemic inflammatory response, and vascular dysfunction is considered as the main pathway (Figure 1). Moreover, the study based on a total of 45 epidemiological cohort data confirmed the areas with the greatest health impact of particulate matter on cardiovascular disease as an increase in mortality due to cardiovascular disease and coronary artery disease. (Figure 2).

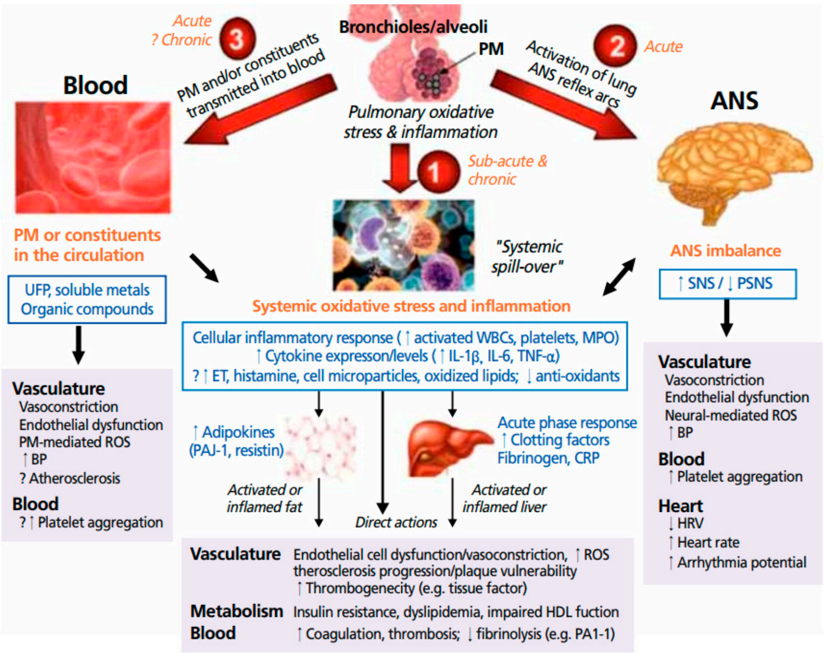

Figure 1. Particulate matter air pollution and cardiovascular disease: an update to the scientific statement from the American Heart Association [Brook, 2010 Circulation]

Health Outcomes	Short-Term Exposure (Days)	Longer-Term Exposure (Months to Years)
Clinical cardiovascular end points from epidemiological studies at ambient pollution concentrations		
Cardiovascular mortality	↑ ↑ ↑	↑ ↑ ↑
Cardiovascular hospitalizations	↑ ↑ ↑	↑
Ischemic heart disease*	↑ ↑ ↑	↑ ↑ ↑
Heart failure*	↑ ↑	↑
Ischemic stroke*	↑ ↑	↑
Vascular diseases	I ↑	↑ +
Cardiac arrhythmia/cardiac arrest	↑	↑
Subclinical cardiovascular end points and/or surrogate measures in human studies		
Surrogate markers of atherosclerosis	N/A	↑
Systemic inflammation	↑ ↑	↑
Systemic oxidative stress	↑	
Endothelial cell activation/ blood coagulation	↑ ↑	↑
Vascular/endothelial dysfunction	↑ ↑	
BP	↑ ↑	
Altered HRV	↑ ↑ ↑	↑
Cardiac ischemia	↑	
Arrhythmias	↑	

Figure 2. Causal relationship between particulate matter and disease, 'Integrated Science Assessment for Particulate Matter' of US Environmental Protection Agency (EPA)

Although the government's policy to reduce emissions of air-pollutants are a fundamental measure to reduce the incidence of air pollution-mediated health problems, the challenges associated with a rapid transition from a fossil fuel-based economy to one that is clean power-based are not achievable in the short term as they related to the structure of the industry.

So, the concentration of indoor PM 2.5 can have a more important health effect on CAD patients than that of outdoor PM 2.5, given that the majority of the population spends more time indoors than outdoors, and the sources of indoor pollutants might differ from those of outdoor pollutants, especially indoor burning of fossil fuels (such as cooking oil).

Portable or central home air-filtration systems have been shown to reduce indoor PM2.5 levels by 50–60% and are more convenient than the use of face masks, allowing use for longer durations. Therefore personalized mitigation measures using portable air-purifier can effectively reduce an exposure to PM2.5 and improve some biomarkers of cardiometabolic health.

B. Study Purpose and Objectives

i. Study Purpose

This research was set as the goal to achieve the following two core research contents.

- Development of clinical research protocol to evaluate the intervention effect of air-purifiers in patients with cardiovascular disease
- Changes in hemodynamic surrogate measures through air-purifier intervention in patients with cardiovascular disease

ii. Study Objectives

We aimed to evaluate the improvement of hemodynamic surrogate measures through air-purifier intervention in CAD patients with PCI

4. Methods

A. Study Participants, Design and Recruitment

i. Investigation Sites

Cardiovascular department of Chungbuk National University Hospital and Chungnam National University Hospital in South Korea will be invited to participate in the study.

Investigators will be selected from the cardiovascular specialty and will agree to comply with all aspects of the investigational protocol.

ii. Study Population

The trial will be conducted in 40 randomized CAD patients with PCI.

Eligible patients will be included in the study as they become available. The order of intervention (active vs. sham filtration) will be randomly assigned. Patients will remain blinded to their treatment assignment throughout the study.

Subjects who sign an informed consent and satisfy all pre-operative inclusion criteria will be considered for randomization and treatment. Consented subjects who do not satisfy entry criteria are screen failures and will not receive intervention under this protocol. Those screen failures will conclude their participation in the study without further follow-up or data collection activities.

iii. Study Design

This is a multicenter, randomized, single-blind (subject), 2-period crossover study design. The study consisted of two intervention periods of 2 weeks separated by a washout period of 2 weeks (Figure3).

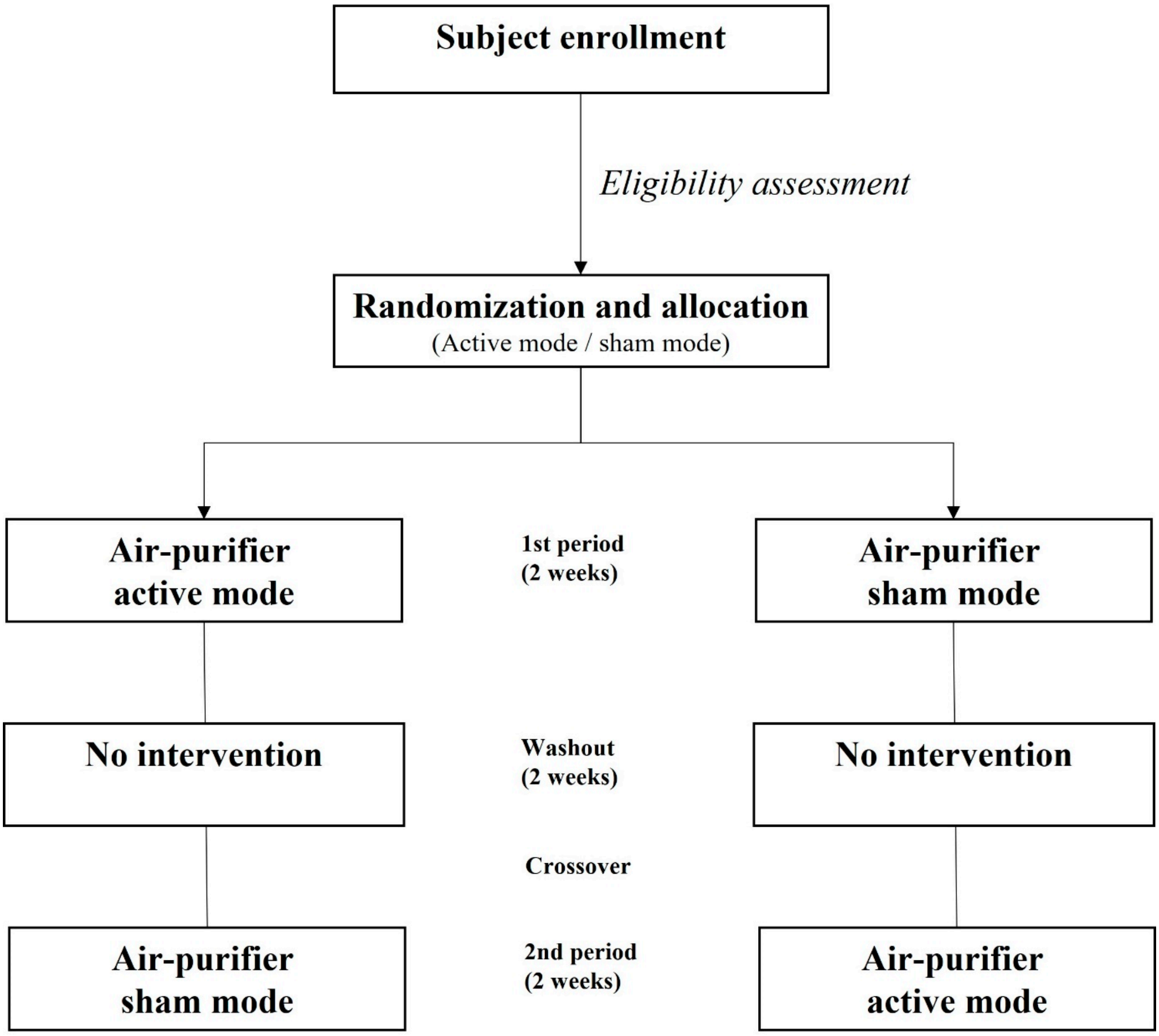

Figure 3. Study design

iv. Sample Size Measures

A sample size of 40 participants was needed to provide 80% power to detect a 4.8% of difference in diastolic blood pressure with the proposed crossover design.

v. Recruitment Procedures

The medical staff of the research participating institutions plan to confirm the intention to participate after explaining the study design directly to patients who can participate in the study.

vi. Patient Withdrawal

All patients are free to withdraw from participation in this study at any time, for any reason, and without prejudice. Patients may be withdrawn from the study for any of the following reasons:

1) Patient requests early discontinuation
2) Patient is lost to follow-up

The clinical investigator may terminate a patient from the study at any time for lack of therapeutic effect in case that is intolerable to the patient, or otherwise considered unacceptable, for intolerable or unacceptable AEs, intercurrent illness, noncompliance with study procedures, administrative reasons, or in the clinical investigator's opinion, to protect the patient's best interest.

Patients who request early discontinuation will be considered as having withdrawn consent only after they have signed the withdrawal of consent form. Patients who withdraw consent will receive standard of care treatment.

The clinical investigator will make every effort to collect information about patients who are lost to follow-up. If a patient is withdrawn before completing the study, the reason for withdrawal (assuming the patient is willing and able to share this information) will be entered on the Study Completion Form. Patients who withdraw before completing the study will be followed only through the date of their withdrawal.

In addition, any comments (spontaneous or elicited) or complaints made by the patient or physician caring for the patient but not involved in the investigation will be documented on the eCRF.

B. Criteria for Eligibility

i. Inclusion criteria

A patient will be eligible for study participation if he/she meets the following criteria:

1) Patient underwent PCI due to coronary artery disease
2) Patient is 18 years of age or older
3) Patients is 85 years old or younger
4) Patient is willing and capable of complying unassisted with the study protocol

requirements including all specified follow-up visits

5) Patient provides written informed consent prior to enrollment in the study

ii. Exclusion criteria

A patient will be excluded from the study if he/she meets any of the following criteria:

1) Patient complaints of dyspnea of functional class IV
2) Patient has LVEF < 45%
3) Patient has decreased CrCl less than 15ml/min
4) Patient has chronic obstructive pulmonary disease (Stage III-IV)
5) Patient underwent PCI within 3 months of enrollment
6) Patient experienced malignant arrhythmia (e.g., ventricular tachycardia or ventricular fibrillation) within 3 months of enrollment
7) Patient experienced cerebral infarction or cerebral hemorrhage within 3 months of enrollment

C. Intervention

Place the air-purifier in the center of the living room and operate the air-purifier continuously for 2 weeks. Air-purifier (model name: ACK 13OZOSKBR) is HEPA filter type (H13), and purifies indoor air at a rate of 324m^2/h.

Recruited eligible patients will be randomly assigned to two intervention sequence (A: active-mode or B: sham-mode), AB and BA, where the patients in the sequence AB assign active-mode (A: with HEPA filter) first, followed by sham-mode (B: without HEPA filter), and vice versa in the BA sequence.

D. Indoor air quality measurement

During the full study period (6 weeks), indoor air quality indices (i.e., PM2.5 and PM10) will be measured using an IoT-based indoor air quality monitoring system (ADT-1783, Smart-Aircok, Korea)

E. Study endpoints

The following study endpoints will be evaluated for all patients who satisfy entry criteria, are randomized, and are subsequently enrolled into the study.

i. Primary Endpoints (Effectiveness)

The primary effectiveness endpoint of the study is a superiority comparison of intervention between air-purifier and sham mode evaluated by followings:

1) Oxidative stress biomarker
2) Endothelial function
3) Heart rate variability
4) Baroreflex sensitivity

ii. Secondary Endpoints

The secondary effectiveness endpoint of the study is a superiority comparison of intervention between air-purifier and sham mode evaluated by followings:

1) Blood pressure
2) Heart rate

F. Schedule of Activities

	Baseline	Intervention	Washout	Intervention
Informed consent	X			
Inclusion / Exclusion	X			
Medical history	X			
Physical examination, height weight	X			

Transthoracic echocardiography	X			
Biomarker*	X	X	X	X
Flow-mediated endothelial function test*	X	X	X	X
Heart rate variability*	X	X	X	X
Baroreflex sensitivity*	X	X	X	X
Vital sign*	X	X	X	X
Particulate matter concentration measurement†	X	X	X	X

* All parameters were measured on the last day of each period

† Particulate matter was continuously measured for 3 days at baseline and 6 weeks at each intervention and washout period.

G. Assignment Consideration

i. Allocation and Sequent Generation

Recruited eligible patients will be randomly assigned to one of the intervention sequences [(A: active-mode or B: sham-mode), AB and BA] using a 2*2 Latin-square design. The patients in the sequence AB assign active-mode (A: with HEPA filter) first, followed by sham-mode (B: without HEPA filter), and vice versa in the BA sequence.

ii. Blinding

All patients will be blinded to the intervention assignment. All patients use an air-purifier at home during the entire intervention period, excluding the washout period. Patients were instructed not to touch the air-purifier, and apparently did not know the difference between active and inactive modes.

H. Study Procedures

i. Screening and Informed Consent Procedures

When a suitable candidate presents for consideration for enrollment in the study, the investigator will explain the potential risks and benefits of participation to the patient. Patients will be provided with a copy of the informed consent for review and will be given ample opportunity to read and pose questions they may have

about the study. If the patient agrees to participate, the informed consent will be signed by the patient and recorded on a screening/enrollment log. If a patient subsequently fails to meet eligibility criteria, they will be considered a screening failure and may receive standard of care.

ii. Pre-intervention Procedures

1) Baseline Evaluations

The following baseline evaluations will be performed for eligible patients within 30 days (or as indicated) prior to the planned intervention

- Written informed consent
- Evaluation of inclusion/exclusion criteria
- Environmental exposure-related evaluation
- Medical history, including demographics, cardiovascular risk factors, cardiac medication
- General physical examination including blood pressure, heart rate, height and weight
- Determination of the NYHA functional class
- Transthoracic echocardiography
- Heart rate variability assessment
- Baroreflex sensitivity assessment
- Flow-mediated dilation assessment
- Blood sampling (biomarkers)
- PM 2.5 measurement sensor installation (Indoor)

Indoor PM 2.5 measurement sensor will be installed in the living room at least 3 days before the intervention period is initiated.

iii. Intervention Procedure

1) Intervention

We will operate the air-purifier (active mode vs. sham mode) in patient's living room for two intervention periods of 2 weeks each. According to the crossover designed allocation table, the intervention is first in active mode and then in inactive mode, or vice versa.

2) Evaluations

We will work on the following evaluation on the last day of each patient's intervention

- General physical examination including blood pressure, heart rate height and weight
- Heart rate variability assessment
- Baroreflex sensitivity assessment
- Flow-mediated dilation assessment
- Blood sampling (biomarkers)
- PM 2.5 measurement (Indoor)

  Indoor PM 2.5 will be continuously measured in the living room for 2 weeks.

iv. Wash-out procedure

- All intervention with air-purifier including sham mode will be stopped for 2 weeks.
- Evaluations

We will work on the following evaluation on the last day of each patient's wash-out period

- General physical examination including blood pressure, heart rate height and weight
- Heart rate variability assessment
- Baroreflex sensitivity assessment
- Flow-mediated dilation assessment
- Blood sampling (biomarkers)

- PM 2.5 measurement (Indoor)

  Indoor PM 2.5 will be continuously measured in the living room for 2 weeks.

v. **Measurement protocol**

- Exposure measurements

During the full study period (6 weeks), indoor PM2.5 concentration will be measured using an IoT-based indoor air quality monitoring system (ADT-1783, Smart-Aircok, Korea). Continuous measurement of indoor PM2.5 (every 1 minute) will be collected from main micro-environment inside the patient's house where patients stay (e.g., living room).

- Health parameter measurements

Depending on the patient's visit time and the order of the examination, the results of the measurements may vary. Therefore, the patient's visit time is adjusted so that the patient visits at the same time.

- Precautions before measurements

Research nurses call the patient 48 hours before the measurements to remind them of the following precautions:

  - There must be no acute illness within 48 hours prior to the test.
  - Antihistamines and alpha-blockers for benign prostatic hyperplasia should be stopped at least 48 hours before.
  - Anti-inflammatory analgesics, including narcotics, should be stopped only in the morning of the test day.
  - Avoidance of strenuous exercise for 24 hours before the test and coffee or cigarettes 6 hours before the test.
  - Fasting after midnight for the test scheduled in the morning, or skip lunch with light breakfast allowed for the test scheduled in the afternoon.

- Measurement sequence

The order of the test should be following;

1) Blood pressure measurement

- After arriving at the examination room, rest in a sitting position for 5 minutes.
- At this time, sit with feet flat on the floor, and the patient rests on a chair with a back to rest.
- Place the upper arm at the level of the heart.
- Choose a cuff that can cover 22-24cm in length, 40% of the circumference of the upper arm, and 80-100% of the circumference of the upper arm.
- Measure twice using a mercury sphygmomanometer.
- Interpretation of results: Check the average value of the systolic/diastolic blood pressure measured twice as the blood pressure

2) Autonomic nervous function tests

After the rest for 5 minutes, patients are administered Finapres Nova (V1.9.A.R5503) for the continuous measurement of heart rate and blood pressure.

(1) Deep breathing test

- After inducing a constant respiration, record the reference value for 1 minute. Record 8 regular breaths at a breathing rate of 6 breaths per minute (inhale 5 sec, exhale 5 sec). After the first test, take a break for 2 minutes, and then proceed with the second test in the same way.

(2) Valsalva maneuver

- In a 30°-tilted position, let the subject practice at least one Valsalva maneuver for seconds. The subject can monitor his expiratory pressure using a mouthpiece connected to the pressure gauge.
- After approximately 1 minute to be relaxed, the subject is instructed to take deep breath and blow into the mouthpiece, keeping the pressure 40mmHg for 15 seconds.

- Repeat Valsalva maneuver twice, total 3 times, with 3 minutes of intervals.
- Select the most representative maneuver for evaluation.

(3) Head-up tilt test

- Get the baseline blood pressure from the brachial artery in supine position.
- Acquire the 5-10 minutes baseline
- Tilt patient up at 70 degree. The transition from supine to tilt position should smooth and of duration 5-10 seconds.
- Typical duration of the tilt should be 10 minutes. Observe subject for the presence of any discomfort, chest pain, shortness of breath, dizziness, lightheadedness, syncope.
- Tilt the patient back.

3) Flow-mediated vasodilation

Vascular endothelial cell function to evaluate vasodilation of brachial artery using ultrasound. Before starting the test, the subject should be sufficiently rested for 10 minutes in a supine position.

(1) Endothelium-dependent vasodilation measurement

- Acquire images from the right brachial artery using a high-resolution ultrasound transducer (10.0 MHz linea-array transducer).

- Obtain a baseline image of the brachial artery from the anterior part of the antecubital fossa.

- For the image of the artery, select the one that shows the clearest intima layer before and after the center along the long axis, align the center and mark it on the skin.

- Obtain a reference image here and measure the arterial blood flow velocity using a pulsed Doppler at an angle of 70 degrees to the blood vessel.

- Calculate the blood flow using the inner diameter area and the blood flow velocity.

- After wrapping the cuff around the upper arm, inflate the cuff with a

pressure of 250 mmHg for 5 minutes to block blood flow to the upper arm and then relax the cuff.

- After relaxation, obtain images of the brachial artery every 15 seconds.

- Use the result measured at 60 seconds.

(2) Endothelium-independent vasodilation measurement

- After resting for about 15 minutes before the test, the brachial artery returns to its baseline state, and then measures the basic diameter and blood flow velocity of the brachial artery.

- In order to check the endothelial cell-independent vasodilation reaction, nitroglycerin 0.6mg NTG is administered sublingually, and the diameter of the blood vessel and the blood flow rate are measured 3 minutes later.

4) Measurements of the autonomic nervous system function test

(1) Cross-correlation baroreflex sensitivity (xBRS)

Cross-correlation and regression between systolic blood pressure and R-R interval are computed over 10 s sliding windows, a time-span sufficient to accommodate fully a 10 s variability in rhythm, or several cycles at ventilator frequencies. In this study, the data will be analyzed with Novascope.

- Parasympathetic function

① Respiratory sinus arrhythmia (RSA)

- RSA is calculated from the average of the consecutive differences between the end of expiration and end of inspiration in heart rate.

② Cardiovagal barosensivitity (BRSv)

- Figure 4 shows the systolic blood pressure during the Valsalva maneuver. BRSv is measured as the regression slope where the systolic blood pressure falls per millisecond (ms) in early phase II (figure 4).

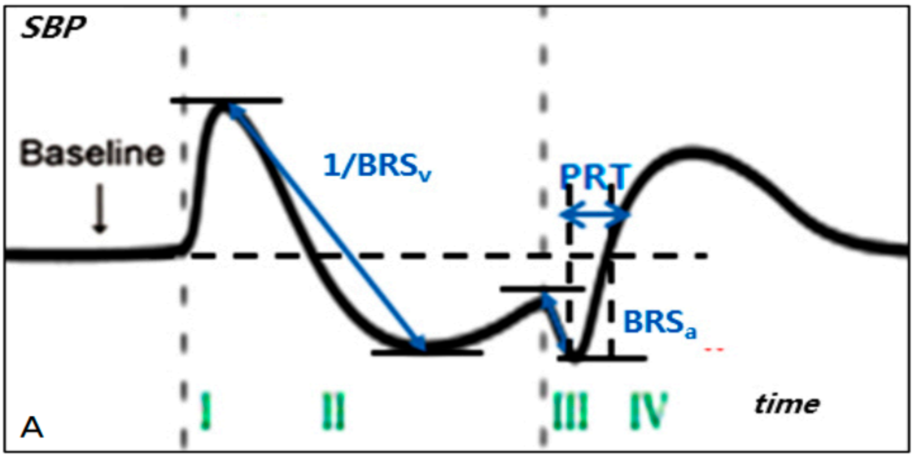

[figure 4]

➢ Sympathetic function

① Pressure recovery time (PRT): PRT was measured as the time (ms) it took for the lowest systolic blood pressure in phase III to return to the baseline blood pressure level.

② Adrenergic barosensitivity (BRSa): The degree of drop in systolic blood pressure in phase III was calculated as the value divided by PRT (Figure 5).

③ Sympathetic index 1 (SI1): This is a picture of the systolic blood pressure during the phase II period.

④ Sympathetic index 3 (SI3): It was measured as the difference between the systolic blood pressure at the end of phase II and the baseline systolic blood pressure.

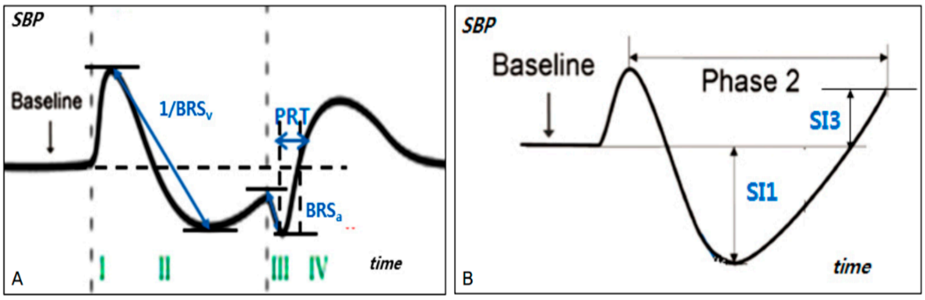

[Figure 5]

5) Blood sample for the biomarkers.

- Blood and urine samples are collected after blood pressure

measurement and autonomic nervous system examination are completed.

- A portion of the blood sample is sent directly to Green Cross for the determination of C-reactive protein (CRP), Interleukin-6 (IL-6) and Brain-Natriurectic Peptide (BNP). Blood samples and urine samples are stored at the Human Resources Bank of Chungbuk National University Hospital and Chungnam National University Hospital, and urine samples are re-sold just before the measurement of urinary 8-OHdG, urine MDA, and urine creatinine.

- Remaining blood and urine samples are stored in the Human Resources Bank for three years and, if necessary, discarded after the expiration of the period of use.

I. Data Collection, management, and Analysis

The study described in this CIP will be implemented according to the requirement of IRB in CBNUH and CNUH. Steps to assure the accuracy and reliability of data include the selection of qualified clinical investigators and appropriate study sites, review of CIP procedures with the clinical investigator and associated personnel prior to the study, and periodic monitoring visits by research nurse.

➢ Data Collection (Case report forms)

The electronic case report forms (eCRFs) contain confidential material. Specific training on the completion of eCRFs will be provided to the clinical investigator and other site. The clinical investigator is responsible for the accuracy and completeness of data reported on eCRFs.

➢ Data Management

A comprehensive data management plan will be developed including a Data Management Overview, Database Contents, annotated eCRF, Pre-Entry Review List, Self-evident Correction Conventions, Query Contacts, and Consistency Checks.

➢ Data Monitoring

Research nurse will conduct site visits to the study facilities to monitor the study

and ensure applicable regulations and guidelines.

The clinical investigator agrees to allow these monitors and access to the clinical supplies dispensing and storage area and to study documentation for the above mentioned purpose and agrees to assist the monitors in their activities, if requested. Requests by regulatory agencies to inspect the study sites may be made. The clinical investigator agrees to allow inspectors from regulatory agencies to review records and to assist the inspectors in their duties, if requested.

The clinical investigator agrees to make source documents (hard copy or electronic) available for this purpose. It is the clinical investigator's responsibility to ensure accurate completion of the eCRFs and to approve the eCRFs. The clinical investigator or a designated sub investigator recognized by the IEC or IRB has the authority to sign eCRFs. These electronic signatures serve to attest that the information contained in the eCRF is accurate and true.

- Statistical Methods

Paired t-test will be used to compare the means of evaluation variable. Effect of intervention will be assessed using a mixed-model analysis. All p-values were two-sided and the statistical significance was set at <0.05. Statistical analysis will be performed using SAS 9.4.

5. Patient Benefits and Risks

i. Potential Benefits to Study Subjects

Receiving benefit from participation in the study is not guaranteed. Anticipated benefits to patients may include, but are not limited to, the following:

- Patients who participate in the study may receive more frequent and/or detailed follow-up than is normally prescribed as standard of care in any given practice
- Overall advancement of medical and scientific knowledge may benefit future patients with similar conditions

ii. Potential Benefits to Study Subjects

It is generally accepted that there are no side effects from the use of the HEPA Filter air-purifier. However, possible risks enrolled in this study include all those risks currently associated with the evaluation methods for hemodynamic indicators in this study. The risks of the evalutation process are related primarily to hypotension. We listed SAE and the other AEs in Table 1.

iii. Clinical Risk Analysis

- Increased Risk to Subjects Posed by the Investigation

All patients who are enrolled in the study will undergo pre-procedure testing that is considered standard of care at the participating institution. The investigator is obliged per protocol to perform the intervention procedure with air-purifier in a manner consistent with their standard clinical practice. However, it is generally accepted HEPA filter air-purifiers do not have any health hazard effect. Therefore, subjects will be randomized to receive treatment with air-purifier without any special concerns about the occurrence of clinical health risks.

At all participating sites, evaluation process required per protocol are consistent with standard protocol of evaluation. The risks associated with follow up evaluation are the same as those for the baseline evaluation procedure. For evaluation procedures, the exposure to vasodilation agents (nitrates) during flow mediated dilation evaluation and sudden change of posture during Tilt table test can pose a potential adverse events

6. Adverse Events

i. Definitions

(1) Serious Adverse Events

Primary Serious Adverse events	Definition
Loss of consciousness	Loss of consciousness refers to a state in which an individual lacks normal awareness of self and the surrounding environment. The patient is not responsive and will not

	react to any activity or stimulation. Syncope is the medical term for temporary loss of consciousness.

(2) Adverse events

Adverse events	Definitions
Dizziness	Dizziness is a term used to describe a range of sensations, such as feeling faint, woozy, weak or unsteady. Dizziness that creates the false sense that you or your surroundings are spinning or moving is called vertigo.
Nausea	Nausea is stomach discomfort and the sensation of wanting to vomit. Nausea can be a precursor to vomiting the contents of the stomach.
Vomiting	Vomiting, or throwing up, is a forceful discharge of stomach contents. It can be a one-time event linked to something that doesn't settle right in the stomach. Recurrent vomiting may be caused by underlying medical conditions.
Flushing	an extra feeding given to ewes before mating to increase the lambing percentage

7. Ethics

i. Declaration of Helsinki

The study will be conducted according to the guidelines established in the Declaration of Helsinki. Patients will be free to withdraw from the study at any time without prejudice to their subsequent treatment.

ii. Institutional Review Board Approval

This study will be conducted in compliance with the Declaration of Helsinki and its amendments and the applicable regulations of the country in which the study is conducted.

A properly constituted, valid Institutional Review Board (IRB) or Independent Ethics Committee (IEC) must review and approve the CIP, the investigator's informed consent document, and related patient information and recruitment materials before the start of the study.

iii. Informed Consent

Informed consent shall be obtained in writing and documented before a patient is enrolled in the clinical investigation in accordance with the principles of Informed Consent.

It is the responsibility of the clinical investigator to ensure that written informed consent is obtained from the patient (or legally acceptable representative) before any activity or procedure is undertaken that is not part of routine care.

iv. Patients Identification and Confidentiality

Patient identification and confidentiality includes but is not limited to the following:

(1) Patients will be identified on all eCRFs by a unique reference code including the patients' initials.

(2) ECRFs are confidential documents and will only be available to the clinical investigator, the biostatistician, and if requested, to the advisory committee and regulatory authorities. The principal clinical investigator for each center will maintain, as part of the investigation file, a list identifying all patients entered into the trial.

J. Regulatory Requirements

(1) Compliance With Regulations Applicable to Clinical Trials

This clinical study is conducted according to the clinical research plan approved by the Korea Centers for Disease Control and Prevention and the Clinical Trial Review Board (IRB). Any changes to the clinical research protocol should be

discussed between the sponsor and the principal investigator. The researcher must obtain approval from the IRB in advance for any changes to the clinical research protocol, except in cases to immediately prevent harm to the research subjects. If the clinical research protocol is changed and applied before IRB approval in order to prevent immediate harm to the research subjects, the change must be reported to the IRB as soon as possible.

(2) General Considerations

➢ Discontinuation of the Study

There were no specific criteria for study discontinuation in this study.

➢ Use of Information and Publication

The principal investigator is responsible for maintaining and providing basic clinical research documents.

Evidence documents include all observational records, records of clinical research activities, and records related to evaluation of clinical research. Therefore, the supporting documents include all records of all treatments performed based on the clinical research protocol or records.

The clinical research data of a research subject who has provided written consent is entered into the CRF according to the CRF preparation guidelines, and the sponsor or his/her representative conducts the verification of the supporting documents to confirm the completeness of the data and the correspondence with the supporting documents. Data that are missing or inconsistent after verification should be explained. The principal investigator signs the CRF after checking the data entered.

In accordance with Article 15 of the Enforcement Regulations of the Clinical Research Bioethics and Safety Act, the implementing institution must keep clinical research related documents for 5 years from the clinical research completion date.

➢ Report to: Korea Centers for Disease Control and Prevention

➢ Reporting time

➢ Interim report: June or July 2020 (consult with the Korea Centers for

Disease Control and Prevention)

- Final result report: December 2020
- Report

- Individual exposure level to particulate matter

- Effect of reducing exposure level to particulate matter by individuals before and after using an air-purifier

- Hemodynamic indicators

- Analysis of hemodynamic indicators before and after intervention

- Threshold of particulate matter level that induces changes in hemodynamic parameters

- Guidelines for the use of air-purifiers for elderly patients with cardiopulmonary disease

- publication will be published one week before the final result report
