# Supplementary material for: Positive Effect of Air Purifier Intervention on Baroreflex Sensitivity and Biomarkers of Oxidative Stress in Patients with Coronary Artery Disease: A Randomized Crossover Intervention Trial
Source: Int J Environ Res Public Health. 2022 Jun 9;19(12):7078. doi: 10.3390/ijerph19127078 (PMC9223013; doi:10.3390/ijerph19127078)
Supplement: Supplementary file 1 [file ijerph-19-07078-s001.zip › S1 Table.pdf]

Table S1. Baseline characteristics of study participants by intervention sequence.

Variable		Active – sham filtration	Sham - active filtration
Number		19	19
Age	yrs	65.7±6.6	65.9±6.4
Sex	Male : female	6:13	2:17
Height	cm	163.9±9.3	166.2±6.2
Weight	kg	71.4±9.4	66.8±7.5
BMI	kg/m^2	26.6±3.1	24.2±2.7
Clinical diagnosis	STEMI	10 (52.6)	7 (36.8)
	NSTEMI	3 (15.8)	3 (15.8)
	Unstable angina	6 (31.6)	9 (47.4)
No of prior PCI	1	16 (84.2)	18 (94.7)
	2	3 (15.8)	1 (5.3)
Underlying disease	Hypertension	5 (26.3)	8 (42.1)
	Diabetes	6 (31.6)	7 (36.8)
	Dyslipidemia	3 (15.8)	7 (36.8)
	HF	1 (5.3)	1 (5.3)
	TIA	0 (0)	1 (5.3)
Antianginal medication	Beta blocker	11 (57.9)	15 (79)
	ACEI/ARB	10 (52.6)	10 (52.6)
	Nitrate	5 (26.3)	2 (10.5)
Smoking status	Current smokers	5 (26.3)	3 (15.8)
	Secondary smokers	3 (15.8)	10 (52.6)
