# Supplementary material for: Positive Effect of Air Purifier Intervention on Baroreflex Sensitivity and Biomarkers of Oxidative Stress in Patients with Coronary Artery Disease: A Randomized Crossover Intervention Trial
Source: Int J Environ Res Public Health. 2022 Jun 9;19(12):7078. doi: 10.3390/ijerph19127078 (PMC9223013; doi:10.3390/ijerph19127078)
Supplement: Supplementary file 1 [file ijerph-19-07078-s001.zip › S2 Table.pdf]

Table S2. **Changes in heart rate variability, deep breathing test, tilt table test and flow-mediated vasodilation between pre- and post-intervention according to intervention phase.**

	Sham filtration			Active filtration		
	Pre	Post	P-value	Pre	Post	P-value
Heart rate variability						
SDNN (ms)	25.1 (1.6)	26.4 (1.6)	0.520	24.8 (1.8)	29.1 (1.6)	0.015
RMSDD (ms)	17.5 (1.9)	17.4 (1.9)	0.928	17.7 (2.0)	18.4 (1.9)	0.654
HF (ms^2)	51.1 (3.4)	50.0 (3.6)	0.895	51.2 (3.7)	58.6 (4.4)	0.554
HFnorm	37.4 (1.8)	38.3 (1.8)	0.809	36.7 (1.6)	35.9 (1.7)	0.849
LF (ms^2)	71.3 (3.1)	66.7 (3.7)	0.740	77.3 (4.3)	94.0 (3.3)	0.328
LFnorm	52.0 (1.6)	51.2 (1.6)	0.872	54.9 (1.6)	57.8 (1.3)	0.537
LF/HF	1.4 (2.7)	1.3 (2.7)	0.774	1.5 (2.5)	1.6 (2.2)	0.701
Deep breathing test						
E:I ratio	1.13 (0.01)	1.13 (0.01)	0.674	1.13 (0.02)	1.13 (0.01)	0.904
HRDB	7.55 (0.79)	7.42 (0.92)	0.833	7.81 (1.48)	7.50 (0.86)	0.796
Tilt table test						
Max delta[a] SBP (mmHg)	-27.83 (4.13)	-26.14 (3.89)	0.662	-25.44 (3.57)	-34.73 (9.25)	0.115
Delta[b] SBP (mmHg)	-16.94 (5.00)	-9.65 (7.37)	0.375	-16.86 (3.90)	-13.8 (5.24)	0.528
delta[b] DBP (mmHg)	-7.58 (2.19)	-4.26 (2.44)	0.313	-7.62 (1.69)	-6.18 (1.88)	0.395
delta[b] HR (beat/min)	8.27 (1.81)	6.41 (0.98)	0.258	4.54 (1.26)	5.30 (1.38)	0.679
Flow-mediated vasodilation						
Endothelin independent dilatation	11.82 (0.74)	11.89 (0.72)	0.845	11.43 (0.72)	11.86 (0.65)	0.158
Endothelin dependent dilation	17.54 (0.91)	17.78 (0.93)	0.389	17.44 (0.99)	18.08 (0.92)	0.093
