# Supplementary material for: Positive Effect of Air Purifier Intervention on Baroreflex Sensitivity and Biomarkers of Oxidative Stress in Patients with Coronary Artery Disease: A Randomized Crossover Intervention Trial
Source: Int J Environ Res Public Health. 2022 Jun 9;19(12):7078. doi: 10.3390/ijerph19127078 (PMC9223013; doi:10.3390/ijerph19127078)
Supplement: Supplementary file 1 [file ijerph-19-07078-s001.zip › S3 Table.pdf]

Table S3. Differences in health outcomes between pre- and post-intervention according to intervention phase.

	Difference* (SE)		
	Sham filtration	Active filtration	P-value
Blood pressure			
SBP (mmHg)	-1.88 (2.17)	-4.18 (2.61)	0.4854
DBP (mmHg)	0.82 (1.44)	-0.18 (1.48)	0.6382
HR (beats/min)	1.06 (1.11)	1.36 (1.47)	0.8704
Autonomic nerve function			
xBRS (ms/mmHg)	0.78 (0.57)	1.94 (0.91)	0.2924
Inflammation and oxidative stress markers			
CRP (mg/dL)	0.28 (0.33)	0.53 (1.14)	0.8252
IL-6 (pg/mL)	6.33 (4.94)	5.07 (4.71)	0.8644
BNP (pg/mL)	-0.30 (0.53)	0.77 (0.93)	0.1756
8-OHdG (μg/g creatinine)	0.78 (0.40)	0.05 (0.64)	0.3632
Heart rate variability			
SDNN	1.87 (2.73)	3.67 (2.63)	0.6495
RMSDD	0.13 (1.10)	0.20 (2.03)	0.9787
HF	12.23 (15.15)	114.27 (95.51)	0.2515
HFnorm	3.00 (3.89)	-0.90 (3.84)	0.5428
LF	-28.37 (40.55)	-56.13 (113.73)	0.5809
LFnorm	-3.00 (3.89)	0.93 (3.84)	0.5391
LF/HF	-0.32 (0.32)	0.16 (0.50)	0.4657
Deep breathing test			
E:I ratio	-0.60 (2.49)	-0.90 (4.63)	0.9552
HRDB	-0.52 (1.38)	0.42 (1.46)	0.4247
Tilt table test			
delta SBP (mmHg)	-4.85 (4.74)	0.18 (1.92)	0.2917
delta DBP (mmHg)	-3.09 (2.79)	-0.85 (1.38)	0.4384
delta HR (beat/min)	1.52 (1.26)	-0.85 (1.66)	0.3457
Flow-mediated vasodilation			
Endothelin independent dilatation	0.05 (0.36)	0.48 (0.32)	0.3595
Endothelin dependent dilation	0.23 (0.30)	0.70 (0.40)	0.3916

*Difference calculated as post-phase minus pre-phase
