# Supplementary material for: Positive Effect of Air Purifier Intervention on Baroreflex Sensitivity and Biomarkers of Oxidative Stress in Patients with Coronary Artery Disease: A Randomized Crossover Intervention Trial
Source: Int J Environ Res Public Health. 2022 Jun 9;19(12):7078. doi: 10.3390/ijerph19127078 (PMC9223013; doi:10.3390/ijerph19127078)
Supplement: Supplementary file 1 [file ijerph-19-07078-s001.zip › S4 Table.pdf]

Table S4

Outcomes	Intervention (Active vs sham) Percent, %	95% CI	P-value	Indoor $PM_{2.5}$ level (one percent increase) Percent, %	95% CI	P-value
Heart rate variability						
SDNN	9.76	-3.60, 24.97	0.232	-11.20	-28.16, 5.77	0.187
RMSDD	6.40	-10.73, 26.81	0.552	-6.26	-26.46, 13.95	0.531
HF	20.20	-20.97, 82.81	0.461	-11.55	-60.21, 37.10	0.630
HFnorm	-5.75	-23.60, 16.28	0.635	14.47	-9.68, 38.63	0.230
LF	40.72	-4.49, 107.32	0.145	-30.88	-75.41, 13.66	0.166
LFnorm	11.40	-5.39, 31.18	0.270	-10.76	-29.88, 8.36	0.259
LF/HF	18.98	-16.15, 68.83	0.405	-23.89	-64.49, 16.70	0.238
Deep breathing test						
E:I ratio	0.54	-1.12, 2.23	0.588	-0.19	-3.07, 2.69	0.894
HRDB	6.67	-8.54, 24.42	0.478	3.65	-22.75, 30.04	0.777
Tilt table test						
Max delta SBP (mmHg)	12.38	-7.55, 32.3	0.300	-7.11	-30.19, 15.98	0.534
delta SBP (mmHg)	7.36	-15.79, 30.50	0.594	-0.56	-28.31, 27.18	0.967
delta DBP (mmHg)	3.80	-6.19, 13.80	0.524	0.11	-11.80, 12.02	0.985
delta HR (beat/min)	3.61	-0.60, 7.81	0.157	-7.02	-14.01, -0.02	0.049
Flow-mediated vasodilation						
Endothelin independent dilatation	4.31	-2.46, 11.56	0.295	-0.85	-8.77, 7.06	0.828
Endothelin dependent dilation	2.69	-2.95, 8.66	0.432	-2.50	-9.12, 4.13	0.448
